# Final destination of surplus cryopreserved embryos. What decision should be made?

**DOI:** 10.5935/1518-0557.20200085

**Published:** 2021

**Authors:** Carlos Wilson Dala Paula Abreu Abreu, Maria Lúcia Andrade Abreu, Maria Mariana Andrade Abreu, João Pedro Andrade Abreu, Luiz Fernando Cal Silva, Ines Katerina Damasceno Cavallo Cruzeiro, Rui Manuel Lopes Nunes

**Affiliations:** 1 Faculdade de Medicina do Centro Universitário UNIFAMINAS - Muriaé/MG/Brasil; 2 Departamento de Ginecologia e Obstetrícia da Casa de Caridade de Muriaé - Hospital São Paulo - CCMHSP - Muriaé/MG/Brasil; 3 Programa de Residência Médica em Ginecologia e Obstetrícia da Casa de Caridade de Muriaé - Hospital São Paulo - CCMHSP - Muriaé/MG/Brasil; 4 Hospital São Paulo - CCMHSP - Muriaé/MG/Brasil; 5 Clínica MedCenter de Muriaé - MG/Brasil; 6 Núcleo de Saúde da Mulher. Faculdade de Medicina do Centro Universitário UNIFAMINAS - Muriaé/MG/Brasil; 7 Hospital Governador Israel Pinheiro - IPSEMG - Belo Horizonte/MG/Brasil; 8 Faculdade de Medicina do Centro Universitário UNIFAMINAS - Muriaé/MG/Brasil; 9 Faculdade de Medicina da Universidade Federal de Juiz de Fora - UFJF/Brasil; 10 Programa de Residência Médica em Reprodução Assistida da Universidade Federal de Minas Gerais - UFMG/Brasil; 11 Laboratório de Reprodução Humana do Hospital das Clínicas da Universidade Federal de Minas Gerais - UFMG/Brasil; 12 Faculdade de Medicina da Universidade do Porto - FMUP - Porto/Portugal; 13 Department International Network UNESCO Chair in Bioethics

**Keywords:** cryopreservation, embryo, *in vitro* fertilization, ethical, final embryo disposal

## Abstract

**Objective:**

The aim of this study was to evaluate what data exists in the literature about the fate of SCE.

**Methods:**

This is an integrative review of papers published in the last ten years, in the Medline and PubMed databases on the subject.

**Results:**

the studies included in this review demonstrate that the difficulties in defining the fate of surplus cryopreserved embryos are mainly due to the absence of specific legislation in most countries. Sociocultural and demographic factors such as religion, treatment period, ethnicity, income, marital status, economic status and education level are factors that influence the choice of the final destination of ECE. This study demonstrated that in most of the countries evaluated, the law or regulations do not provide clear guidance on the final destination of the surplus cryopreserved embryos, although it is reasonable to consider that the law will treat embryos, after a certain period of time, as abandoned. Accurate information about the desired destination of the surplus cryopreserved embryos is needed from the beginning of the breeding process to minimize future problems.

**Conclusions:**

We conclude that this is a controversial issue, involving ethical, legal, moral and financial issues, that lack specific legislation in almost all of the countries evaluated, which contributes to indecision, abandonment, and it hinders the final destination of these embryos.

## INTRODUCTION

In 1978, in England, the first baby generated by in vitro fertilization (IVF) was born, contributing to the birth of millions of children worldwide. Since then, assisted reproduction techniques have develop and became a reality. With the technique development, the first baby was born from a frozen embryo (cryopreserved) in 1984, in Australia. As a consequence of IVF, more embryos were made, and only a few of them are implanted, and the viable surpluses go into the freezing process. With embryo cryopreservation, it is possible to allow more attempts at pregnancy, reducing the risk of twin pregnancies, limiting the number of embryos transferred. However, a huge supply of stored embryos have been reported worldwide, leading to conflicts and questions about the moral and ethical legality of the fate of these cryopreserved surplus embryos. There are many questions regarding the moral status of the cryopreserved surplus embryos, which in turn raises the question of possible destinations for these embryos, among them: transferring to the mother when she wants to have more children; donate to other patients and/or couples, donate for scientific research or discard (Takahashi *et al*., 2012).

The cryopreservation of gametes or embryos has been an opportunity for young cancer patients to preserve their fertility before starting chemotherapy (Provoost *et al*., 2012; Wånggren *et al*., 2013). In these cases, the procedure presents challenging ethical issues in relation to informed consent, given that the reason that triggered the procedure is a serious disease with an expected degree of mortality. In the midst of controversies, the discussion about the final destination of the SCE gains more importance. Although there are still no precise answers, there are many questions to be considered: is the human embryo produced in vitro life? What to do with the millions of surplus cryopreserved embryos that are in the freezing banks around the world? Keep frozen? Donate them? Discard them? How? Where? The ethical difficulties for final embryo disposal decisions are especially guided by the absence of specific legislation in most countries, on what should be done with the surplus cryopreserved embryos and who is authorized to make the decision (Provoost *et al*., 2012). However, in view of the current scenario and in the absence of a consensus in relation to the various ethical problems that have arisen with the practice of assisted reproduction, in many countries a standardization has started through laws or reference standards.

Determining the effectiveness of the clarifications provided by healthcare professionals during and after IVF treatment regarding couples’ preparation for surplus cryopreserved embryo destination decisions was the subject of a study group at McMaster University, Ontario, Canada, as well as several other centers of research around the world. According to the study, the instructions received after treatment was considered inadequate. Couples with long-term infertility and those in conflict with the final embryo disposal may be appropriate targets for further interventions. More written information and/or post-treatment counseling services can help patients make informed and timely decisions about their surplus embryos (Deniz *et al*., 2016; Fruchter & Shalev, 2015; Jin *et al*., 2012; Lyerly *et al*., 2011; Nachtigall *et al*., 2010; Nelson *et al*., 2008; Scott *et al*., 2012; Souza *et al*., 2018; Takahashi *et al*., 2012).

The decision-making process for unused cryopreserved embryos stored involves a lot of emotional load influenced by sociocultural factors (Souza *et al*., 2018). The main goal of this paper was to run an integrative review of the articles published on the final destination of the excess cryopreserved embryos through the techniques of assisted reproduction in the last 10 years.

## MATERIAL AND METHODS

As for methodology, we used the integrative literature review, which enables the synthesis and analysis of scientific knowledge already produced on the investigated topic (Souza *et al*., 2010).

For this integrative review, we took the following steps: we established the guiding hypothesis and goals; we set the inclusion and exclusion criteria for the papers (sample selection); we defined the information to be extracted from the selected papers and analyzed the results, discussion, results and conclusion of the reviews.

We searched Medline and PubMed banks, using previously defined keywords (cryopreservation and embryo and , assisted reproduction and ethical and embryo end disposition) and the following selection criteria:

1. Full text: available;
2. Main subject: cryopreservation, IVF and embryo destination;
3. Limit: studies carried out in humans;
4. Year of publication: 2010 to 2019.
5. Language: English.

From reading the abstracts and, when necessary, from reading the complete paper, those with more accurate information with the proposed goal were selected.

The results and discussion of the data obtained was done in a descriptive manner, enabling the assessment of the applicability of the integrative review elaborated, in order to achieve the goal in this method.

## RESULTS

Through automated search, where we defined keywords, exclusion and inclusion criteria, we found 85 studies. Of these, 25 met the inclusion criteria. Of the selected articles, most describe studies carried out in assisted reproduction centers, some in a university environment, from different countries and continents, with diverse legal and socio-cultural realities. The final destination of the SCE in most cases is not explicitly determined or specified in the law, when it exists, or in the regulations of the Medical Boards or assisted reproduction committees (Riezzo *et al*., 2016; Scott *et al*., 2012; Souza *et al*., 2018).

Research conducted at the Center for Medical Law and Ethics and the Law School of King’s College London, UK, analyzed elements of the legal consent process for the donation of “spare” embryos for research, including stem cell research, and did a recommendation with the aim of improving the quality of this process, including occasionally, protecting oneself against the invalidity of such consent. We obtained qualitative data from three IVF clinics in the United Kingdom, studying the often delicate and contingent nature of what becomes, for legal purposes, a ‘spare’ embryo. We found that the way in which an embryo becomes ‘spare’, with its implications for the consent process for research donation, is not addressed in the relevant reports or codes of practice governing the donation of embryos for research, which assume a non-problematic notion of the ‘spare’ embryo. And even if, if the quality of that first consent is compromised, it will affect the quality of consent for the donation of this ‘spare’ embryo for research, followed by the quality of consent for future assisted reproduction treatment cycles, as they are needed as a result of a donation decision. This is important to maximize reproductive treatment options for couples involved in IVF treatment and to avoid possible harm to them (Scott *et al*., 2012).

A retrospective study, carried out between 1992 and 2006, with 2,334 couples, from a university center in Belgium, with the objective of evaluating trends regarding the final destination of SCE, showed a positive trend in the donation to science, a fact that opens up more optimistic perspectives for the availability of embryos for stem cell research, and a negative trend in donation to other couples and means of disposal. The positive trend of donation to science, according to the authors, comes from the increase in public awareness of stem cell research, making patients more aware of potential benefits. The study also revealed that a substantial number of patients chose different final destinations for the embryo, at different times of the study, which shows that the advance guidelines, presented before the onset of treatment, should be used with caution (Provoost *et al*., 2012).

A similar study was carried out in Japan, a country of eastern customs and culture, where in 2012 it was estimated that there were approximately 61 million SCE in storage, 15% of which had no defined plans. In Japan, the storage time limit is regulated until the end of the woman’s reproductive life and, at the end of this period, the patient must choose between three options: continue the storage by paying an additional cost, discard, or donate to research. Donation to other infertile people is prohibited. Sociocultural and demographic factors such as religion, treatment period, ethnicity, income, marital status, status and education were reported as factors that influence the donation for research. Ethnic minorities were less willing to donate embryos to research, while most Asians approved the use of embryos for scientific research, unlike Westerners, and in particular Protestants (Takahashi *et al*., 2012).

This topic was also the subject of a study in Canada, in 2013. Most of the evaluated patients prefered to keep the embryos stored, and the rest were divided between donating for research or discarding them. Among those who donated for research or clinical training, three key themes emerged: a desire to “give back”, to contribute to scientific progress and to avoid “wasting” embryos. However, most of these patients were not sure whether they had chosen to donate for research or clinical training. Emphasizing the importance of providing adequate information, that is, reproduction services must ensure that patients are aware of their options regarding the destination of the SCE and that they understand the nature of their options (Provoost *et al*., 2012).

In Canada, according to the Canadian Institutes of Health Research Guidelines and the Assisted Human Reproduction Act, specific consent from gamete and/or embryo donor patients is required before they can be used for research purposes. In order to develop guidelines for physicians participating in the informed choice process in relation to human embryo donation for research purposes, the Canadian Society of Obstetrics and Gynecology Ethics Committee, among other guidelines, determines that the final decision on the SCE donation for research should not be taken until the woman/couple decides she/they no longer need the embryos for reproductive purposes. The decision to end cryopreservation must occur at a different time than the decision concerning the fate of the embryos (Nelson *et al*., 2008).

Patients’ opinions on embryo storage time limits were studied by a group of researchers from Portugal, with interviews conducted from 2011 to 2012, in a single reproduction center, which limits the external validity of the results. It is important to note that in this study, the findings pointed out that patients should be informed of the facts related to cryopreservation, based on a practical ethical reasoning about embryo storage. In general, 38% of the participants preferred the duration of 4-5 years, 38% extended beyond 5 years and 23% indicated 3 years. Having experienced at least one previous cycle was directly associated with agreeing to a storage period of more than 5 years, for both women and men. Having children was inversely associated with longer storage times among women. Although it did not aim to significantly influence the limits followed for storage, this study drew attention to a critical discussion around the need to develop practical guidelines on embryo storage limits (Pereira *et al*., 2015).

In Sweden, where the donation of embryos to other infertile couples was prohibited, a study was carried out in 2013, aiming to find out what were the attitudes towards different aspects of embryo donation among infertile couples who had SCE. The answer was that almost three quarters of the couples interviewed were in favor of embryo donation. Most respondents were also in favor of donating embryos for research. The results indicate that the donation of SCE to other infertile couples in Sweden, would be a common practice if it were an allowed practice, being dependent on a review of the national legislation (Wånggren *et al*., 2013).

A Portuguese study, carried out between 2011 and 2012, that had the objective of evaluating the factors associated with the desire to donate embryos for research, among couples submitted to IVF. This study included 213 heterosexual couples undergoing fertility treatment in a public center in Portugal. The results showed that most couples were willing to donate embryos for research, citing benefits for science, health and infertile patients. Almost all couples reached consensus on the decision. The willingness to donate was more frequent in women under the age of 36 and who considered embryonic research to be very important; and among Catholic men. Those who did not want to donate reported conceptualizing embryos as children or living beings and lack of information, or fears concerning embryo research. Men with higher levels of anxiety were less willing to donate. Therefore, results indicate that future research on this topic should include the assessment of gender differences and psychosocial factors. And, that ethically robust policies and accurate information about research results in human embryos are needed (Samorinha *et al*., 2016).

In 2019, the Scientific Committee of the Reproductive Medicine Society of Argentina published a study demonstrating that Argentina, like many other countries in the region, faces the dilemma of what to do with the growing buildup of cryopreserved embryos, which are often abandoned. It describes that the disposal of embryos is a complex and intimate decision, which depends not only on the quality of the cryopreserved embryo, but also on social, cultural, economic, labor and health aspects. Furthermore, in the absence of a formal regulatory framework for such decisions in Argentina, current practices and standard procedures face significant development obstacles. As a conclusion, we advise that among future actions to be developed in the short, medium and long term by this committee, they are building interdisciplinary teams, promoting patient awareness, developing guidelines and applying policies in relation to embryo abandonment (Lima *et al*., 2019).

A similar study carried out in China, in 2012, on the fate of SCE and embryo donation for medical research, revealed that family size was the main reason for the (dis) continuation of embryonic storage by participants; in addition, the moral status of the embryos was an important factor for couples who choose embryo storage, while the storage rate was an important factor for couples who chose embryo disposal. Most couples stopped embryo storage when their children were over 3 years of age. 58.8% of couples preferred to discard SCE instead of donating it to research, citing lack of information and mistrust in science as significant reasons for their decision. The authors concluded that clarifications about cryopreserved embryos, including patients’ expectations regarding storage and information about SCE, can assist them in making decisions about embryo disposal, thus beneficial policies related to the disposal and donation of embryos in China (Jin *et al*., 2012).

A prospective study, carried out between 2007 and 2010, in France and published in 2016, evaluated 247 IVF patients with surplus cryopreserved embryos who made a final decision on the fate of these embryos. According to the options available, 91 people chose to stop cryopreservation, 77 chose donation for research and 48 donation of embryos for infertile couples. The results obtained after adjusting for age, sex, gamete donation, number of children and the different embryonic representations, an option to stop cryopreservation is more frequent if the embryo is represented as a child. Representing the embryo as a project led patients to choose donation for research. Respondents are more likely to choose embryo donation if they understand the embryo as a potential person. In addition, patients who have benefited from gamete donation are ten times more likely to donate their embryos to another couple. For more than half of the participants (57%), decision making was easy; however, the decision to stop cryopreservation was significantly more difficult than choosing the donation for research or other couples (Bruno *et al*., 2016).

In posthumous assisted reproduction (PAR), discussions about the final disposal of SCE also emerge. To report the results of the analysis of IVF users’ choices regarding the potential of their SCE for PAR, analyzing signed consent forms of 498 patients from a public clinic, Côté *et al*. (2016) demonstrate that most men and women agreed to leave their SCE to their partners for posthumous use in a real life context. However, PAR involves complex issues, including the psychological aspects of starting a pregnancy during grief over the loss of a loved one or the future effect on the child.

## DISCUSSION

The Opinion of the Ethics Committee of the American Society for Reproductive Medicine (ASRM), published in 2013, advises that “programs must create and apply written policies on the designation, retention and elimination of abandoned embryos. Furthermore, in the absence of specific program policies, it is ethically acceptable for a program or establishment to consider that embryos have been abandoned if at least 5 years have passed since contact with an individual or couple, provided that efforts are made to contact the individual or couple, and that there are no written instructions from the couple on disposal. In view of these cases, programs can dispose of embryos by removing storage and thawing without transfer, although in no case can embryos considered abandoned be donated to other couples or used in research ”(Ethics Committee of the American Society for Reproductive Medicine, 2013; Tonkens, 2013).

Cryopreservation and subsequent transfer of SCE is offered as a routine practice in most services in Brazil. For patients, it brings opportunities, along with ethical and emotional challenges related to the need to make a decision, mainly about future transfers, and an embryo’s final destination (Provoost *et al*., 2012).

Dilemmas and ethical questions accompany the lives of those who live with the situation of having SCE. How do people assume that their own genetic material is preserved in the laboratory? Is it biological material or is it a frozen child? Some people, when they have had other children, prefer to discard the remaining embryos because they do not want to donate their genetic material. In addition, there are many embryos left after a couple’s separation, widowhood or city change. When the couple leaves the embryos, is the bank responsible for the maintenance costs, or who will take care of it? What to do with these orphaned embryos? What is allowed to be done? Who decides or what decides their fate?

Another ethical dilemma of cryopreservation is whether to stop a potential person in time, while their parents move forward with each change of the seasons and years. It is difficult to predict what will happen to this couple, family and society during the passage of time. No one is sure of the future of this particular embryo. No one is sure if it will ever be implanted. It is evident that not every embryo will generate a human person. Uncertainties abound.

Questions about the real need to create this entire SCE contingent are wholesome. Although it’s known that nowadays, when one aims blastocyst culture and single embryo transfer, this does not mean we need embryos at the beginning, some countries restrict the number of oocytes to be fertilized per cycle, like what happens in Italy and France, for example. In Italy, the current regulation is very restrictive in this regard. It forbids the use of cryopreserved embryos for purposes different from reproduction, in addition it obliges the assisted human reproduction clinics to provide a long-lasting (virtually everlasting) storage of cryopreserved embryos, also in case of poor-quality embryos (Faustini *et al*., 2019). Likewise, the legislation of Portugal and Brazil does not indicate numbers, but recommends that physicians use reason in the management of reproductive techniques (Pereira *et al*., 2015; Souza *et al*., 2018).

The decision-making process for SCE and unused ones involves a large emotional load influenced by socio-cultural factors. Decisions in this area can be presented at three different levels: the woman or couple seeking treatment, the rules of each institution, and the legislation of the countries. At each of these levels of decision, a necessary position is taken, albeit implicitly, on moral status and respect due to SCE. If this prior definition eludes those who decide the merits of the decision, it will always be weak and, finally, confusing (Beca *et al*., 2016).

When couples definitively decide to interrupt their parenting projects, the symbolic presentation of the embryo remains the main factor that influences the fate of their cryopreserved embryos. In addition, this representation can evolve when influenced by events and external information provided, in addition to the costs involved. To support patients who are making this difficult decision, it may be useful to explore this symbolic representation at the beginning of the IVF procedure, before surplus embryo freezing, as a new tool that improves the accuracy of counseling (Bruno *et al*., 2016).

The question of “abandoned embryos” arises when surplus IVF embryos are cryopreserved and stored for later use and the patient does not return to seek or dispose of these embryos for various reasons. If the fertility clinic or storage facility in question does not have a clear direction on what to do with these embryos and/or payment for storage ceases and/or the patients cannot be reached, the embryos pose an ethical and practical challenge. On the one hand, there is a commitment to respect the autonomy of couples, embryo suppliers, to determine what should be done with the cryopreserved embryos. On the other hand, there are strong reasons why fertility clinics and storage facilities do not want responsibility, potentially perpetually, for other people’s cryopreserved embryos. A Canadian study published in 2016, showed that, despite the country having a complex legislative structure, there are important gaps that leave reproductive services in the tenuous position of discarding “abandoned embryos” without clear authorization, or storing them indefinitely. They concluded that the clarity in the consent procedures, together with flexible deadlines for embryo storage, provide an approach that can better serve the interests of all involved (Cattapan & Baylis, 2016).

In the face of so many controversies there are attempts to reduce the number of embryos to be cryopreserved as much as possible. Once a couple with fertility disorders terminates a pregnancy conquered by transferring cryopreserved embryos, the theme of the remaining embryos available is latent. What to do? Postpone the transfer, donate the embryos to infertile couples, donate for research, discard the embryos.

It is clear the change in attitude of people with SCE in countries where legislation is in place. In Belgium, in 2007, a law came into force that established a maximum shelf life of 5 years (with possible exceptions), forcing patients to make a decision before their first treatment, the tendency to discard decreased and the rates increased donation to others and to science (Provoost *et al*., 2012).

Despite the short physical distance, in Germany, patients receiving fertility treatment appear to be disadvantaged due to the restrictive Embryo Protection Act (“Embryonenschutzgesetz, ESchG”), which does not allow stock fertilization neither selection of the best embryo. Now, the so-called German Middleway (DMW) has been enacted since 2008/2009. It consists of a liberal interpretation of the ESchG in which, according to the couple’s wishes, as many ova as necessary are cultivated beyond the 2 pro nuclear stage to enable identification of 2 viable embryos for selection. This new program can benefit particularly older patients with adequate ovarian reserves (Kliebisch *et al*., 2016).

In Brazil, there is no specific legislation to standardize assisted reproduction techniques, including the destination of SCE. The Federal Board of Medicine (FBM) has been regularly publishing and updating resolutions on assisted reproduction techniques in general. The first FBM resolution on the subject was published in 1992 (Resolution 1358/1992). On September 21, 2017, FCM published its last Resolution to date (Resolution 2168/2017), on assisted reproduction procedures. Regarding the cryopreservation of gametes or embryos (item V), it defines that cryopreserved embryos can be discarded after three years or more, if it is the patients’ expressed will. In addition, for the first time, it claims that this also applies to abandoned embryos. They define it in a single paragraph that abandoned embryos are those that the owners did not comply with the pre-established contract, and these people were not located by the clinic that performed the procedure. According to Souza *et al*. (2018), the logical implication is that we need to discuss and look for reasonable reasons to justify a new design of national policies and practices. Perhaps it is time to also revisit the discussion about the possibility of allocating them to scientific and/or clinical research before their destruction (Souza *et al*., 2018). In March 2005, Biosafety Law 11,105 was enacted in Brazil, which authorizes the use of human embryos, produced through IVF, for the generation of stem cells for research or therapeutic purposes. For this purpose, the embryos must be unfeasible or have been frozen for 3 (three) years or more, on the date of publication of this Law, or which were already frozen on the date of publication of this Law, after completing 3 (three) years, counted from the date of freezing.

As previously discussed, there are few studies in few countries that assess the difficulties and complexities related to the fate of surplus frozen embryos using assisted reproduction techniques. The authors, in general, report that the ethical difficulties in defining the fate of SCE are mainly due to the absence of specific national legislation on the subject. In addition, sociocultural and demographic factors such as religion, treatment period, ethnicity, income, marital status, economic status and education have been reported as factors that influence the final destination of the SCE. Several authors propose that women or couples should have the right to decide independently, while institutions should be clear in their regulations. And legislation must establish the embryo’s legal status before implantation, the rights of couples and the regulation of cryopreservation of the embryo. Personal, institutional or legal decisions must take a view on the moral status of the human embryo, and try to avoid its destruction or indefinite storage.

## CONCLUSION

The decision of each individual must be entirely free, based on their values and criteria, avoiding any kind of advice from the professionals involved. This is showing true respect for the autonomy of their decisions. When deciding, couples should also consider what it means for them to have SCE, taking into account the psychological aspects that involve the issue over time.

Fertility centers and ethics committees are responsible for discussing these issues proactively, so that comprehensive consent recommendations can be implemented. However, it is clear that as long as there is no legal rule imposing limits, the problems of doubts and uncertainties about the legal status of these embryos will remain.

Due to the uncertainties of these situations, the programs must require that each individual or couple, holders of SCE, have written instructions on the final disposal in the event of death, divorce, separation, inability to bear the storage charges, inability to dispose in the future, or prolonged lack of contact with the program. This written decision should be in existence before the first treatment cycle, or at the moment of cryopreservation (Ethics Committee of the American Society for Reproductive Medicine, 2013).

In cases where written instructions for the final disposal of the embryos do not exist, and the individual or couple cannot be located, the program will be faced with the possibility of indefinite storage or the elimination of embryos, absorbing their costs. Currently, in most countries, the law does not provide clear guidance on when it is lawful to discard abandoned embryos, although it is reasonable to consider that the law will treat embryos, after a certain period of time, as abandoned. In the face of legal uncertainty, some programs prefer to continue storing embryos abandoned indefinitely. Other programs assume the risk in the final disposal of the SCE and abandon them, after a long period of storage and without contact with the right holders, in the decision for the destination of the embryos, as directed by the FBM (Souza *et al*., 2018).

Thus, precise information about the SCE destination policy is necessary, adequate clarification of couples/patients and signature of free and informed consent prior to freezing about the possible destinations of frozen embryos. However, the ethical and moral discussion is wide, it involves many variables and should always be encouraged.
